# Atrial Fibrillation: Prevalence and Association With Outcome in Patients With Stroke Undergoing Mechanical Thrombectomy in the United States

**DOI:** 10.1161/SVIN.123.001248

**Published:** 2024-07-02

**Authors:** Fadar Oliver Otite, Smit D. Patel, Haydn Hoffman, Ehimen Aneni, Nnabuchi Anikpezie, Emmanuel Oladele Akano, Claribel Wee, Devin Burke, Karen Albright, Timothy Beutler, Julius Gene Latorre, Ashish Sonig, Amit Singla, Nicholas Morris, Seemant Chaturvedi, Priyank Khandelwal

**Affiliations:** ^1^ Department of Neurology State University of New York Upstate Medical University Syracuse NY; ^2^ Department of Neurosurgery University of Connecticut Hartford CT; ^3^ Department of Neurosurgery State University of New York Upstate Medical University Syracuse NY; ^4^ Section of Cardiovascular Medicine Department of Internal Medicine, Yale University School of Medicine New Haven CT; ^5^ Department of Population Health University of Mississippi Medical Center Jackson MS; ^6^ Molecular Neuropharmacology Unit National Institutes of Neurologic Disorders and Stroke, NIH Bethesda MD; ^7^ Department of Neurosurgery Rutgers University Newark NJ; ^8^ Department of Neurology University of Maryland School of Medicine Baltimore MD

**Keywords:** atrial fibrillation, epidemiology, ischemic stroke, mortality

## Abstract

**Background:**

How the prevalence of atrial fibrillation (AF) has changed over time in various demographic subgroups of patients with acute ischemic stroke (AIS) undergoing mechanical thrombectomy (MT) in the United States is unknown. Whether in‐hospital outcomes differ between patients with AF versus patients without AF after MT remains uncertain.

**Methods:**

We conducted a serial cross‐sectional study using all primary AIS discharges in the 2010 to 2020 National Inpatient Sample. Discharges with MT codes were identified (n = 155 277), and the proportion with AF in various age, sex, and racial subgroups were computed. We used multivariable‐adjusted negative binomial regression to compare AF prevalence between demographic subgroups and joinpoint regression to evaluate trends over time. Multivariable‐adjusted generalized linear models were used to evaluate the association of AF with in‐hospital outcomes.

**Results:**

Across the study period, 45.0% of AIS discharges with MT had AF, but prevalence varied by age, sex, and race or ethnicity. After multivariable adjustment, AF prevalence was 4% higher in women versus men (prevalence rate ratio, 1.04 [95% CI, 1.01–1.07]) and was lower in Black versus White (prevalence rate ratio, 0.80 [95% CI, 0.77–0.84]) but higher in Asian compared with White discharges (prevalence rate ratio, 1.11 [95% CI, 1.05–1.18]). Prevalence increased with age (prevalence rate ratio for ≥80 years versus 18–39 years, 5.23 [95% CI, 4.28–6.39]). Following joinpoint regression, prevalence increased by 3.2% (95% CI, 1.3%–5.2%) annually across the period 2010 to 2015 but declined by −2.2% (95% CI −2.9% to −1.4%) from 2015 to 2020. AF was associated with 22% lower odds of in‐hospital death (odds ratio, 0.78 [95% CI, 0.71–0.85]) and 13% greater odds of routine home discharge (odds ratio, 1.13 95% CI, 1.04–1.22]) compared with no AF.

**Conclusion:**

AF prevalence in patients undergoing MT in the United States is approximately twice that of the general AIS population. AF prevalence in MT increased from 2010 to 2015 but declined from 2015 to 2020. In the subset of patients with AIS undergoing MT, AF is associated with reduced in‐hospital death.

**Nonstandard Abbreviations and Acronyms**

AIS: acute ischemic stroke
HERMES: Highly Effective Reperfusion Evaluated in Multiple Endovascular Stroke Trials
HCUP: Healthcare Cost and Utilization Project
LVO: large‐vessel occlusion
NIS: National Inpatient Sample
MT: mechanical thrombectomy
STAR: Stroke Thrombectomy and Aneurysm Registry

**Clinical Perspective**

**What Is New?**

- Approximately 45% of hospitalizations for mechanical thrombectomy in acute ischemic stroke (have comorbid atrial fibrillation (AF), and this proportion increases with age, with 70% of patients aged ≥80 years having AF.
- Compared with non‐AF admissions, AF is associated with reduced in‐hospital death and better odds of routine home discharge.

**What Are the Clinical Implications?**

- In contrast with all patients with acute ischemic stroke, in the subset of patients with acute ischemic stroke undergoing mechanical thrombectomy, AF may be associated with better short‐term outcomes.

Atrial fibrillation (AF) is the most common sustained arrhythmia^1^ and is associated with increased risk of acute ischemic stroke (AIS).^2^ More than 20% of all AIS admissions in the United States have comorbid AF,^3^ but prevalence estimates in AIS with large‐vessel occlusion (LVO) may likely be higher.^4^ Whereas mechanical thrombectomy (MT) is now guideline‐recommended therapy for AIS with LVO and its use has increased significantly in the United States over the past decade,^5^ how AF prevalence varies between various demographic subgroups of these MT‐treated LVOs is not known. AF prevalence increases with age^6^ and may disproportionately affect men compared with women. However, women with AF have greater stroke risk,^7^ particularly those aged >80 years.^8^ Black people may also have lower prevalence of AF compared with White people,^9^ but the age or sex groups of MT‐treated LVOs in which these racial differences in prevalence may be most noticeable remain unidentified.

With aging of the population,^10^ AF prevalence in MT‐treated AIS over the past decade may have increased. However, advances in primary care and AF treatment over this time period, including more frequent use of any or direct oral anticoagulants in the United States over the past decade,^11^ may have led to fewer patients with AF presenting with LVO. Thus, it remains unknown how AF prevalence in patients undergoing MT has changed over time.

Furthermore, compared with non‐AF AIS, AF‐associated AIS has been shown to carry a more dismal outcome.^12^ Infarct size tends to be larger and hemorrhagic transformation risk typically greater in AF‐associated AIS compared with non‐AF AIS.^12^ However, whether outcomes differ between patients with AF and patients without AF in the subset of patients with LVO undergoing MT remains controversial.^4^, ^13^

The primary aim of this study is to (1) obtain age‐, sex‐, and race or ethnicity‐specific prevalence of AF in patients with LVO undergoing MT; (2) evaluate trends in the prevalence of AF in MT‐treated LVOs in the United States over the past decade; and (3) compare in‐hospital death and odds of routine home discharge between AF and non‐AF associated AIS in the United States.

## Methods

### Standard Protocol Approvals and Data Availability

This study was conducted using the National Inpatient Sample (NIS), one of the data sets from the Healthcare Cost and Utilization Project (HCUP). According to HCUP, use of the deidentified NIS does not require an institutional review board review. The NIS is publicly available for direct purchase from HCUP. The authors are bound by data use agreement not to share HCUP data, but data analysis codes used in this study can be made readily available to interested parties on reasonable request. This study was conducted in accordance with the Reporting of Studies Conducted Using Observational Routinely Collected Data guidelines.^14^ The first author (F.O.O.) was responsible for data stewardship.

We used data contained in the 2010 to 2020 NIS to conduct a serial cross‐sectional study. The NIS is the largest publicly available inpatient care database in the United States and consists of a 20% stratified random sample of all acute inpatient discharges in US hospitals. Further details on the NIS are available at hcup‐us.ahrq.gov.

### Study Population

We identified all adults (aged ≥18 years) primary AIS discharges by querying the NIS using *International Classification of Diseases*, *Ninth Revision* (*ICD‐9*) codes 433.X1, 434.XX, and 436 before October 2015 and *ICD*, *Tenth Revision* (*ICD‐10*) codes I63.XX afterward. These codes have been validated previously and found to be concordant with physician‐diagnosed AIS in >90% of cases.^15^ To minimize the risk of double counting hospitalizations, we excluded all discharges with length of stay of <24 hours and discharge to another short‐term/acute hospital. All elective discharges were also excluded.

### Definition of Covariates

Discharges with diagnostic codes for MT were defined using ICD‐9 Clinical Modification (*ICD‐9‐CM*) procedure code 39.74 and *ICD‐10 Clinical Modification* (*ICD‐10‐CM*) codes 03CG3ZZ, 03CG3Z6, 03CG3Z7, 03CG4Z6, 03CG4ZZ, 03CH3ZZ, 03CH3Z7, 03CJ3ZZ, 03CJ3Z7, 03CK3ZZ, 03CK3Z7, 03CL3ZZ, 03CL3Z7, 03CP3ZZ, 03CP3Z7, 03CQ3ZZ, and 03CQ3Z7. AF was defined using *ICD‐9‐CM* codes 427.3x and *ICD‐10* codes in the range of I48.xx. These codes have been validated previously and shown to have high accuracy for AF.^16^, ^17^, ^18^, ^19^ Hospitalizations with intravenous thrombolysis were defined using *ICD‐9‐CM* procedure code 99.10 or *ICD‐10‐CM* code 3E03317. We also classified discharges as those for intravenous thrombolysis if they had *ICD‐9‐CM* code V45.88 and *ICD‐10‐CM* code Z92.82, corresponding to IV‐tPA administration within 24 hours at an outside facility. Furthermore, we reviewed the discharge Medicare Severity Diagnosis Related Group code and categorized admissions under the intravenous thrombolysis if they had Medicare Severity Diagnosis Related Group codes in the range of 061–063 (for “ischemic stroke with thrombolytic agents with or without comorbidity/conditions or major complication/comorbidity”). Admissions with secondary intracerebral hemorrhage were identified using secondary discharge codes in the range of 430, 431, or 432.9 (*ICD‐9*) or *ICD‐10* codes I60–I62.

We used the “Elixhauser” add‐on package in Stata to compute the Elixhauser score, a validated comorbidity score consisting of 31 comorbidities for all patients.^20^ The National Institutes of Health Stroke Scale (NIHSS) was defined using *ICD‐10* codes R29.7xx. Baseline CHA_2_DS_2_‐VASc of all admissions were computed using components of the score (Table S1). race or ethnicity and sex were determined using the HCUP variables “RACE” and “FEMALE.” Further descriptions of these variables are available at https://hcup‐us.ahrq.gov/db/vars/race/nisnote.jsp and https://hcup‐us.ahrq.gov/db/vars/female/nisnote.jsp.

In‐hospital all‐cause death was defined using the HCUP variable “DIED.” We defined good outcome as routine home discharge, and this was defined using the HCUP variable “DISPUNIFORM.” In‐hospital length of stay was determined using the HCUP variable “LOS,” and total charges for each discharge were determined using the variable “TOTCHG.”

### Statistical Analysis

We summarized baseline characteristics of MT‐treated AIS with and without AF using descriptive statistics. Sampling weights provided in the NIS allow for estimation of national estimates on the basis of the 20% of admissions included in the NIS.^21^ We used these weights to compute the weighted prevalence of AF in MT admission subgroups categorized by age, sex, and race or ethnicity. A statistical test of differences in prevalence between demographic subgroups was evaluated using the Pearson χ^2^ test. We further used negative binomial regression adjusted for differences in demographic factors, and clinical and hospital‐level variables to compare the prevalence rate of AF between demographic subgroups.

We also computed the annual prevalence of AF in MT‐treated AIS and used joinpoint regression to evaluate trends in prevalence over time. Joinpoint uses a series of Monte Carlo–based simulations to identify points of change in trends (joinpoints) in a data set. A regression line to the natural logarithm of the rates using calendar year as the regressor variable is then fitted to compute annualized percentage change for each identified trend.

We further used generalized linear models adjusted for demographic factors, stroke severity (NIHSS), Elixhauser score (comorbidity burden), and other hospitalization factors to evaluate differences in odds of in‐hospital death between hospitalizations with prevalent AF and those without. The Elixhauser score used in these models was modified to exclude cardiac arrhythmias to avoid double adjustment for AF. Similar models were also used to evaluate the odds of secondary intracerebral hemorrhage or routine home discharge in hospitalizations with AF compared with those without. Other generalized linear models were used to evaluate the association of AF with in‐hospital length of stay or total charges. Independent variables in multivariable models were selected a priori on the basis of their likely association with AF or AF detection or with hospitalization outcome. Because of the potentially strong confounding effect between stroke severity and these outcomes, we evaluated these outcomes only in admissions with available NIHSS. However, we conducted additional analyses in all patients with and without this variable but excluding this variable from the multivariable model. We considered the weighting and clustering needed in the complex NIS survey data analysis. To account for changes in the NIS survey design in 2012, we used relevant NIS “TRENDWT” as recommended by HCUP. Because this is a descriptive analysis with no specific analysis tested, adjustment for multiple comparisons was not necessary. A *P* value of <0.05 was required for statistical significance in all analyses. All analyses were done by the first author (F.O.O.) using Stata 16 (StataCorp, College Station, TX) and Joinpoint software version 4.8.0.1 (Bethesda, MD).

### Missing Variables

The *ICD‐10* code for NIHSS in the administrative database was introduced in 2016 and was available in 64.9% of MT admissions from 2016 to 2020. Most other variables except race or ethnicity (5.4%) were missing in <2.5% of admissions. Admissions with missing race or ethnicity were categorized into an unknown category. Admissions with missing information on other variables were imputed to the dominant category. Missing insurance data were categorized into the Medicare category if age ≥65 years and into the dominant category otherwise. For the NIHSS, multivariable models were conducted in all patients but not including the NIHSS and in additional models restricted only to patients with available scores.

## Results

Of 392 302 031 weighted hospital discharges in the United States from 2010 to 2020, the prevalence of comorbid AF in these discharges increased from 9.4% in 2010 to 13.8% in 2020 (Figure S1). Among the 5 190 148 primary AIS admissions in the United States over this time, 3.0% (n = 155 277) underwent MT. A total of 45.0% of these MT admissions had comorbid AF. Baseline characteristics of MT admissions with and without AF are summarized in Table 1. Patients admitted for MT with AF were relatively older and more likely to be White individuals (Table 1). Baseline CHA_2_DS_2_‐VASc scores, NIHSS, and Elixhauser comorbidity scores were significantly higher in AF compared with non‐AF admissions (Table 1). The proportion of AF admissions with codes for intravenous thrombolysis (36.2%) and for mechanical ventilation (24.8%) were significantly lower compared with non‐AF admissions (39.5% and 26.0%, respectively; *P*<0.0001).

**Table 1:** Baseline Characteristics of Mechanical Thrombectomy Discharges in the United States From 2010 to 2020 According to Atrial Fibrillation Status

Variables	No atrial fibrillation	Atrial fibrillation	*P* value
Total number^*^	17 128	13 989	N/A
Weighted number ^†^ (%)	85 469 (55.0)	69 808 (45.0)	N/A
Female, %	45.5	56.5	<0.0001
Age, y, mean ±SD	63.4 ±14.6	75.3 ±11.4	<0.0001
Age, %			<0.0001
18–39 y	6.5	0.8	
40–59 y	31.8	8.9	
60–79 y	47.1	48.4	
≥80 y	14.7	41.9	
Race or ethnicity, %			
White	62.6	69.5	<0.0001
Black	17.1	10.1	
Hispanic	8.2	7.9	
Asian/Pacific Islander	2.8	3.9	
Other/unknown^‡^	9.3	8.6	
Insurance status, %			<0.0001
Medicare	48.3	76.3	
Medicaid	13.3	5.3	
Private insurance	29.5	14.4	
Self‐pay	5.7	2.2	
Other	3.2	1.8	
Clinical factors, %			
Hypertension	74.5	85.4	<0.0001
Diabetes	27.3	29.1	0.0013
Dyslipidemia	51.4	56.9	<0.0001
Coronary artery disease	23.0	31.6	<0.0001
Congestive heart failure	17.4	34.6	<0.0001
Dementia	4.5	9.6	<0.0001
Intravenous thrombolysis	39.5	36.2	<0.0001
Mechanical ventilation	26.0	24.8	0.0214
NIHSS, mean ±SD	14.6 ±7.7	16.3 ±7.7	<0.0001
Elixhauser comorbidity score, mean ±SD	4.3 ±1.9	5.7 ±1.9	<0.0001
Modified Elixhauser score, mean ±SD^§^	4.2 ±1.9	4.7 ±1.9	<0.001
CHADS‐VASc, mean ±SD	2.9 ±1.7	4.1 ±1.6	<0.0001
Hospital characteristics, %			
Hospital region			<0.0001
Northeast	17.1	18.2	
Midwest	21.5	21.3	
South	40.9	36.8	
West	20.4	23.6	
Hospital location/teaching status			0.0136
Rural	0.3	0.2	
Urban nonteaching	8.5	9.5	
Urban teaching	91.2	90.3	
In‐hospital outcomes			
Length of stay, d	8.8	8.3	<0.001
Total charges, $	201 577	191 283	<0.001
In‐hospital death	13.2	13.0	0.4957
Routine home discharge	14.2	22.9	<0.0001

N/A indicates not applicable; and NIHSS, National Institutes of Health Stroke Scale.

* Represents actual number of admissions contained in the National Inpatient Sample.

^†^ Represents projected national ischemic stroke admission estimates rounded to the nearest whole number, obtained after applying sampling weights to the National Inpatient Sample data set.

^‡^ Other/unknown group includes Native American, Multiple Races, Unknown or Not provided race.

^§^ Modified Elixhauser score represents Elixhauser score with atrial fibrillation excluded.

### Age and Sex Differences in Prevalence

**Figure 1:**
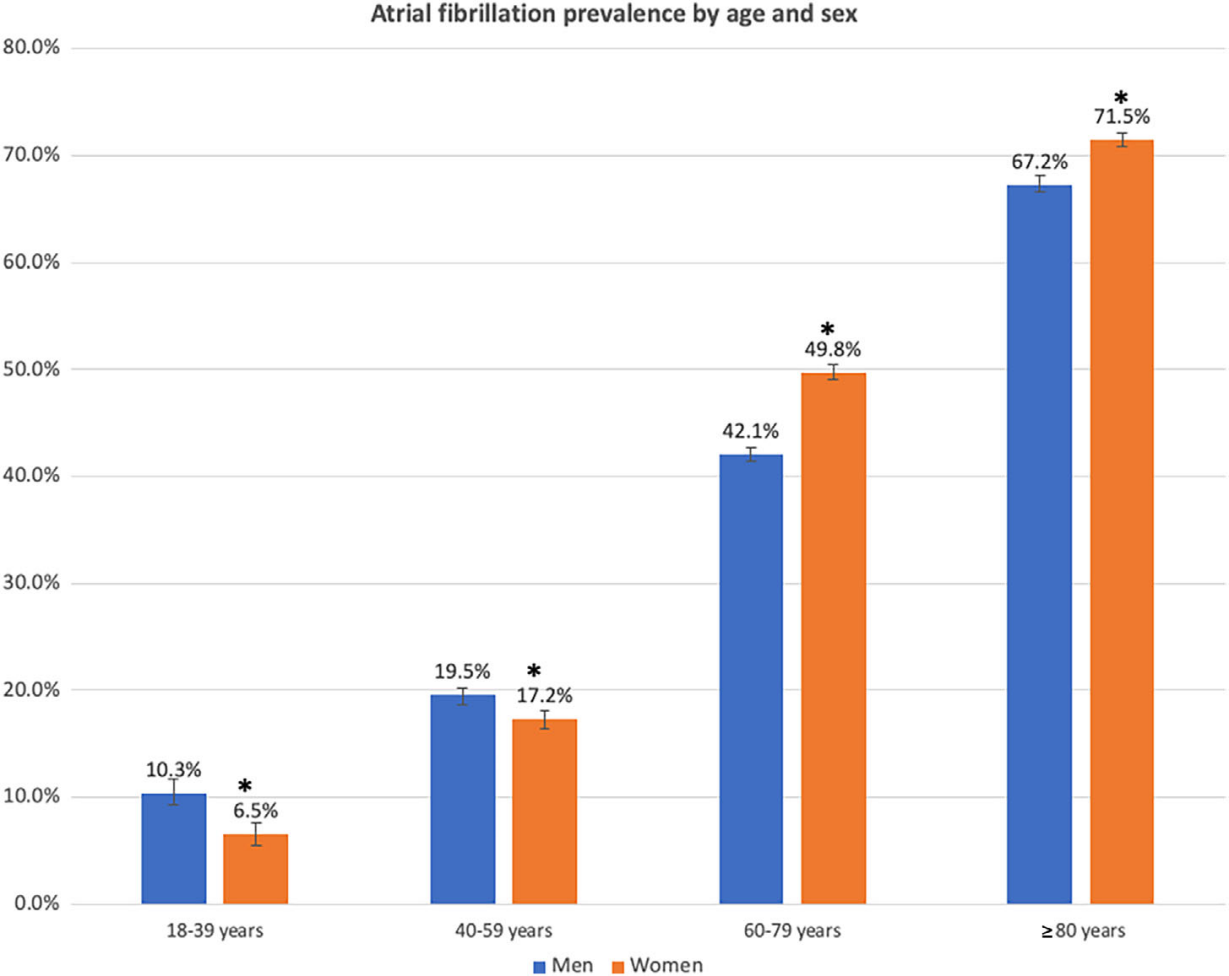
**Prevalence of atrial fibrillation in mechanical thrombectomy discharges in the United States according to age and sex**. *Indicates *P* value <0.05 for comparison between men and women. Error bars represent standard error.

AF prevalence in MT increased by a factor >1.5‐fold with each 20‐year increase in age, such that 70.0% of individuals aged ≥80 years had comorbid AF (Figure 1). Overall AF prevalence was significantly greater in women (50.4%) compared with men (39.5%; *P*<0.0001), but upon stratification by age, the age‐specific prevalence was significantly higher in men in all age groups <60 years, but greater in women compared with men in MT admissions aged ≥60 years (Figure 1).

### Racial Differences in Prevalence

**Figure 2:**
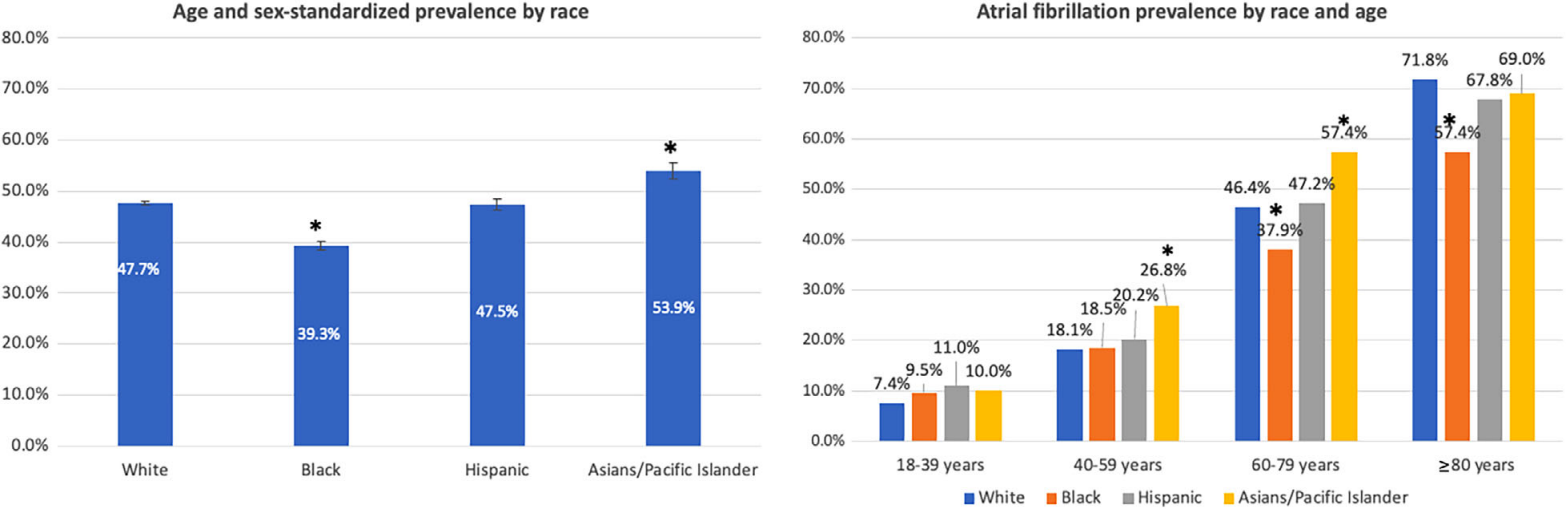
**Prevalence of atrial fibrillation in mechanical thrombectomy discharges in the United States according to age and race or ethnicity**. *Indicates *P* value <0.05 for comparison of racial estimate to that of White admissions (in the same age group in age‐stratified estimates). Absence of * indicates *P* value for comparison >0.05.

Unadjusted prevalence of AF was greater in admissions in White people (47.7%) compared with Black admissions (39.3%). This difference in prevalence was driven by markedly higher prevalence in White admissions in people aged 60 to 79 years and ≥80 years, as there was no significant difference in prevalence between Black admissions and White admissions in age groups <60 years (Figure 2). In contrast, unadjusted AF prevalence in MT admissions in Asian people was significantly greater compared with prevalence in White admissions, primarily due to higher AF prevalence in admissions in individuals aged 40to 59 and 60 to 79 years (Figure 2).

### Multivariable‐Adjusted Difference in Prevalence Between Demographic Subgroups

After multivariable adjustment for age, other demographic, clinical, and hospital‐level factors, AF prevalence in MT admissions in Black people was 20% less compared with prevalence in White people (prevalence rate ratio, 0.80 [95% CI, 0.76–0.84]), but prevalence in Asian admissions was 11% greater compared with White admissions (prevalence rate ratio, 1.11 [95% CI, 1.05–1.18]; Table 2). Prevalence in women was 4% greater than prevalence in men (prevalence rate ratio, 1.04 [95% CI, 1.01–1.07]) and increased with age. Prevalence of AF in patients aged ≥80 years was >5 times the prevalence in young individuals aged 18 to 39 years (Table 2).

**Table 2:** Multivariable‐Adjusted Comparison of the Prevalence of Atrial Fibrillation in Mechanical Thrombectomy Ischemic Stroke Discharges in the United States from 2010 to 2020

Variables	Prevalence ratio^*^	95% CI	*P* value
Race or ethnicity			
Black vs White	0.80	0.77–0.84	<0.001
Hispanic vs White	0.97	0.92–1.01	0.176
Asian/Pacific Islander vs White	1.11	1.05–1.18	<0.001
Women vs men	1.04	1.01–1.07	0.005
Age group			
40–59 vs 18–39 y	2.0	1.7–2.5	<0.001
60–79 vs 18–39 y	3.96	3.25–4.83	<0.001
≥80 vs 18–39 y	5.23	4.28–6.39	<0.001

* Model further adjusted for income, hospital region, hospital location/teaching status, smoking, dyslipidemia, dementia, smoking status, intravenous thrombolysis, CHA_2_DS_2_‐VASc score, and year.

### Trends in Prevalence Over Time

**Figure 3:**
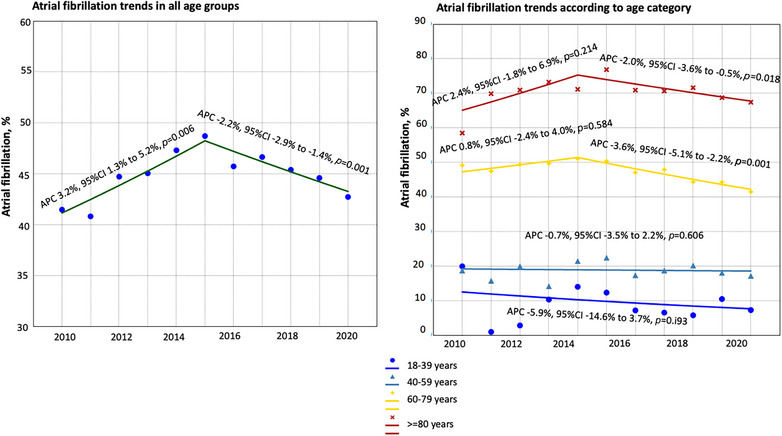
**Trends in the prevalence of atrial fibrillation in mechanical thrombectomy discharges in the United States from 2010 to 2020**. APC represents annualized percentage change.

After joinpoint regression, AF prevalence increased by 3.2% annually over the period 2010 to 2015 (annualized percentage change, 3.2% [95% CI, 1.3%–5.2%]; *P* = 0.006) but declined by ≈2% across the period 2015 to 2020 (annualized percentage change, −2.2% [95% CI, −2.9% to −1.4%]; *P* = 0.001; Figure 3). Most of the initial increase in AF prevalence and subsequent decline across the study period occurred in admissions in patients aged 60 to 79 years and ≥80 years, as the prevalence in younger patients did not change significantly across the study period (Figure 3). Age and sex‐adjusted prevalence also declined in Black and White discharges across the period 2015 to 2020 (Figure S2).

### Multivariable Association of AF With Secondary Intracerebral Hemorrhage, In‐Hospital Death, and Odds of Routine Home Discharge

In generalized linear models, AF was associated with reduced odds of in‐hospital death in nested models adjustment for age differences alone (odds ratio, 0.80 [95% CI, 0.75–0.96]; Table 3, model 2) and in multivariable models that were adjusted further for relevant demographic and clinical factors but not including the NIHSS (odds ratio, 0.78 [95% CI, 0.72–0.85]; Table 3, model 3). Multivariable models with further adjustment for the NIHSS in the subset of admissions with available NIHSS (Table 3, model 4) also showed an association of AF with lower odds of all‐cause in‐hospital death (odds ratio, 0.72 [95% CI, 0.62–0.83]). AF was also associated with greater odds of routine home discharge after multivariable adjustment (odds ratio, 1.13 [95% CI, 1.04–1.23]). Odds of secondary intracerebral hemorrhage and mean length of stay did not differ between hospitalizations with and without comorbid AF, but mean total hospital charges were at least $5000 less in AF compared with non‐AF discharges (Table 3).

**Table 3:** Association of Atrial Fibrillation With Odds of In‐Hospital Outcomes Following Mechanical Thrombectomy

Variables	Model estimates^*^	95% CI	*P* value
Model 1 – unadjusted model (weighted n = 155 277)			
Intracerebral hemorrhage	1.08	1.02 to 1.15	0.005
Length‐of‐stay, d	−0.49	−0.72 to −0.26	<0.001
Total charges, $	−$10 294.02	−14332.72 to −6255.33	<0.001
In‐hospital death	1.02	0.96 to 1.10	0.496
Routine home discharge	0.56	0.52 to 0.59	<0.001
Model 2 (model 1 plus further adjustment for age) (weighted n = 155 277)			
Intracerebral hemorrhage	1.08	1.01 to 1.14	0.020
Length‐of‐stay, d	0.25	0.02 to 0.48	0.030
Total charges, $	263.39	−4092.72 to 4619.49	0.906
In‐hospital death	0.80	0.75 to 0.86	<0.001
Routine home discharge	0.96	0.90 to 1.04	0.310
Model 3 (model 2 plus variables in footnotes) (weighted n = 155 277)			
Intracerebral hemorrhage	1.06	0.99 to 1.13	0.075
Length‐of‐stay, d	0.03	−0.13 to 0.18	0.750
Total charges, $	−$5168.91	−8056.31 to −2281.51	<0.001
In‐hospital death	0.78	0.71 to 0.85	<0.001
Routine home discharge	1.13	1.04 to 1.22	0.003
Model 4: Model 3 plus further adjustment for NIHSS but restricted only to those with available score (weighted n = 78 790)			
Intracerebral hemorrhage	1.09	0.99 to 1.19	0.076
Length of stay, d	−0.12	−0.33 to 0.07	0.211
Total charges, $	−$6602.31	−10 851.67 to −2352.95	<0.001
In‐hospital death	0.72	0.62 to 0.83	<0.001
Routine home discharge	1.16	1.04 to 1.29	<0.001

Model 1: unadjusted model comparing atrial fibrillation admissions to non–atrial fibrillation admissions. Model 2: model 1 plus adjustment for age. Model 3: model 2 plus further adjustment for age, sex, race or ethnicity, insurance status, intravenous thrombolysis, smoking status, dementia, Do‐Not‐Resuscitate, modified Elixhauser score (excluding atrial fibrillation), mechanical ventilation, coma/stupor, dyslipidemia, hospital region, hospital location/teaching status, year of discharge. Model 4: model 3 plus NIHSS. Model restricted to admissions with available NIHSS. NIHSS indicates National Institutes of Health Stroke Scale.

* Estimates represent odds ratio for dichotomous variables (intracerebral hemorrhage, in‐hospital death, and routine home discharge) and β coefficient for continuous variables.

## Discussion

In this contemporary NIS analysis, we found that 45.0% of MT admissions in the United States had comorbid AF, which is approximately twice the prevalence in the general AIS population. However, marked variation in prevalence existed by age, sex, and race or ethnicity. After multivariable adjustment, prevalence increased with age and was marginally higher in women compared with men. Prevalence was ≈20% higher in White compared with Black admissions, but 10% lower in White when compared with Asian/Pacific Islander admissions. Overall AF prevalence increased in the first half of the decade from 2010 to 2015 but declined significantly in the latter half from 2015 to 2020 mainly in patients aged ≥60 years. MT admissions with comorbid AF had ≈20% lower odds of all‐cause in‐hospital death and 10% greater odds of routine home discharge compared with those without AF.

Accurate understanding of demographic variations and trends in AF prevalence is critical for discerning changes in the epidemiologic characteristics and outcome of LVO AIS. Multiple prior MT studies have reported crude AF prevalence in MT, but large‐scale studies systematically evaluating its prevalence in various demographic subgroups are hitherto lacking. Among all participants enrolled in MT trials contained in the HERMES (Highly Effective Reperfusion Evaluated in Multiple Endovascular Stroke Trials) collaboration meta‐analysis, the prevalence of AF was 33%.^22^ However, the clinical characteristics of these highly selected clinical trial patients may not be generalizable to all patients undergoing MT in the real world. In another meta‐analysis of studies evaluating outcomes in patients with AF undergoing MT, the prevalence of AF in the 10 included studies ranged from 20% to 55%.^4^ In this study, we provide new information highlighting AF prevalence in various age, sex, and racial groups that are generalizable to all patients undergoing MT in the United States.

Although it has been well established that AF prevalence increases with age,^6^ the exceptionally high prevalence in patients aged ≥80 years (70.0%) in this study is notable and implies that clinicians taking care of elderly patients undergoing MT may need to consider a more rigorous search for underlying AF in this age group when the source of a suspected embolic stroke remains unidentified. Further subgroup analysis of our data revealed sex differences in AF prevalence with increasing age. Whereas AF was more prevalent in men in admissions of patients aged <60 years, prevalence was greater in women in admissions of patients aged ≥60 years. AF in women carries a higher AIS risk compared with men,^7^ but this excess risk may not be apparent in individuals aged <75 years.^23^ Other major causes of LVO AIS such as extracranial carotid atherosclerosis^24^ may be more prominent causes of AIS in men compared with women. All these factors may potentially contribute to the higher AF prevalence noted in the elderly women undergoing MT compared with similar aged men. In addition, the underuse of anticoagulants in women^25^ may lead to presentation with an AF‐related AIS.

Our finding of higher prevalence of AF in White compared with Black admissions is consistent with previously known racial differences in community‐wide AF prevalence. However, the higher prevalence of AF in Asian/Pacific Islander admissions compared with White admissions undergoing MT is surprising partly because AF is less prevalent in the general population^9^ and in AIS in Asian compared with White people.^26^ Moreover, intracranial atherosclerotic disease may account for up to half of all AIS in Asian people,^27^ so one would expect intracranial atherosclerotic disease to account for a disproportionately greater proportion of LVO in Asian admissions in this study. Whether Asian/Pacific Islander people with AF in the United States are more prone to LVO stroke compared with other races or ethnicities or how racial or ethnic differences in detected AF may be contributing to these differences.^28^ It is also possible that the relatively higher AF in White compared with most minority racial and ethnic groups may be biased by racial disparity in MT access.

In this study, we report on the prevalence of AF but are unable to ascertain a pathogenetic relationship between AF and AIS. We are also unable to differentiate between AF present before admission or AF detected after stroke. AF detected after stroke may represent a subset of AF that may carry lower AIS recurrent risk.^29^, ^30^ Nevertheless, given that the CHA_2_DS_2_‐VASc scores of all these patients with AF will be no less than 2 after stroke, our findings imply that almost half of all patients undergoing MT in the United States may be potential candidates for therapeutic anticoagulation on the basis of current guidelines.^31^ In elderly patients aged ≥80 years, this proportion may even be higher: every 7 in 10.

The rising prevalence of AF in MT in the period 2010 to 2015 is consistent with data from our prior study and those of others reporting increased prevalence of AF in all AIS in the United States over the decade from 2003 to 2014.^3^, ^32^ However, the decline in AF prevalence noted over the period 2015 to 2020 is also consistent with another of our more recent studies demonstrating plateauing prevalence of AF in all AIS admissions in the United States over the same period.^33^ This decline was despite an observed increase in AF prevalence in all hospitalizations over the study period. It is likely that concerted public health efforts toward ensuring appropriate primary stroke prevention in individuals with AF through more frequent use of therapeutic anticoagulation may be playing a major role in this decline. More widespread usage of non–vitamin K antagonist oral anticoagulants and other measures for secondary stroke prevention in patients with AF such as left atrial appendage closure may likely be playing a role. However, there is still considerable room for improvement in use of direct oral anticoagulants for stroke prevention in AF.^34^ Future studies targeted at understanding these changes are needed.

Prior studies evaluating the association of AF with outcomes following MT have yielded conflicting results. Whereas the HERMES collaboration meta‐analysis of MT randomized trials found no difference in odds of modified Rankin Scale score of 0 to 2 at 90 days between patients with AF and patients without AF,^35^ another meta‐analysis of 11 randomized trials reported a positive association between AF and good outcome.^13^ A more recent meta‐analysis of 10 retrospective observational studies reported 47% greater odds of 90‐day death in AF compared with patients without AF,^4^ but this study did not account for difference in age between patients with AF and patients without AF,^4^ a major confounder of its association with death. All of these studies are small in comparison with the current study and recruited patients mainly from large academic centers that may not be reflective of the total MT population in the United States. That AF is associated with increased death and poorer functional outcome compared with other AIS subtypes has been established in multiple cohorts.^36^, ^37^, ^38^ Our data, however, suggest that in the subset of patients with AIS undergoing MT, AF may be associated with lower odds of in‐hospital death and better odds of routine home discharge. In‐hospital length of stay is similar but corresponding charges of hospital stay are significantly less in discharges of patients with AF compared with patients without AF undergoing MT. The lower mortality odds was seen in models adjusted for age alone, despite patients with AF presenting with more severe strokes as measured by the NIHSS. The exact underlying reasons for this possibly better outcome in AF MT admissions still requires further exploration in prospective registries, but it is possible that faster MT procedural times in patients with AF, an important determinant of MT outcomes, may potentially be responsible for this. In one recent analysis of 4169 patients undergoing MT contained in the STAR (Stroke Thrombectomy and Aneurysm Registry), procedural times were faster, MT passes were fewer, and rates of first‐pass success were higher in AF compared with non‐AF admissions.^39^ Moreover, some forms of non‐AF LVO, such as those due to underlying intracranial atherosclerotic disease, carry high risk of MT failure, defined as Thrombectomy in Cerebral Infarction grade <2,^40^ and MT failure may be associated with larger AIS core and worse functional outcome compared with AF‐associated strokes.^40^

This study has additional limitations. Although the ICD codes used for AIS and AF have been shown to have high accuracy, we cannot exclude coding errors. Our study of trends in AF prevalence over time is based on the implicit assumption that coding practices remained unchanged over time. However, improved coding is likely to have led to increased AF documentation and prevalence over time, yet we observe a decline over the period 2015 to 2020. The number of codes per encounter retained in administrative data sets such as the NIS has increased over time,^17^ and this is expected to also lead to increased prevalence of comorbidities, not the decline we are noting for AF. In fact, AF in all hospitalizations in the NIS also increased over the years 2010 to 2020. Our study period bridges that between *ICD‐9* and *ICD‐10*, and this may be a potential source of coding error. The general algorithm to categorize AF did not change from *ICD‐9* to *ICD‐10*,^17^ so no major difference in AF prevalence is likely to be attributable to changes in *ICD* coding. Our evaluation of secondary intracerebral hemorrhage should be viewed with caution, as the sensitivity of *ICD‐10* codes for this complication is still unknown.

This study likely underestimates the true burden of AF in MT, as a significant proportion of paroxysmal AF continues to be diagnosed beyond AIS hospitalization. We have no specific information on the location of LVO, timing from presentation to recanalization, or the degree of recanalization, all of which may potentially be associated with AIS outcome. Methods for obtaining race and ethnicity data in the NIS is not standardized across hospitals or states and is not always based on the gold standard of self‐report. We have no information on nonbinary gender expressions because these are set to missing in the NIS.

## Conclusion

Of all hospitalizations in patients with AIS undergoing MT in the United States over the decade from 2010 to 2020, 45.0% had comorbid AF, and this proportion increased with age, varied with sex, and was disproportionately higher in Asian people compared with White and in White compared with Black discharges. Prevalence increased over time from 2010 to 2015 and declined from 2015 to 2020. AF is associated with reduced odds of in‐hospital death in admissions of patients with AIS undergoing MT.

## Sources of Funding

None.

## Disclosures

Dr Chaturvedi is an associate editor for the *Stroke* journal and Dr Otite is on the *Stroke* journal editorial board. The other authors have no relevant disclosures.

## Supporting information


**Table S1**. International Classification of Diseases Codes for identifying covariates.
**Figure S1**. Trends in the prevalence of atrial fibrillation in all admissions in the United States from 2010‐2020 regardless of diagnoses
**Figure S2**. Trends in age‐ and sex‐adjusted prevalence of atrial fibrillation in acute ischemic stroke admissions by race.

## Data Availability

All datasets used in this study are publicly available for purchase from the Healthcare Coste and Utilization Project (HCUP). Data analysis codes may be shared upon reasonable request but the authors are bound by datause agreement not to share purchased HCUP data.
