# Supplementary material for: Atrial Fibrillation: Prevalence and Association With Outcome in Patients With Stroke Undergoing Mechanical Thrombectomy in the United States
Source: Stroke Vasc Interv Neurol. 2024 Jul 2;4(5):e001248. doi: 10.1161/SVIN.123.001248 (PMC12778550; doi:10.1161/SVIN.123.001248)

Table S1. International Classification of Diseases Codes for identifying covariates

Variable	International Classification of Diseases Ninth revision codes	International Classification of Diseases Tenth revision codes
Hypertension	Elixhauser comorbidity software	Elixhauser comorbidity software
Diabetes mellitus	Elixhauser comorbidity software	Elixhauser comorbidity software
Dyslipidemia	HCUP CCS software code 53	HCUP CCS software code 53
Coronary artery disease	HCUP CCS code 101	HCUP CCS code 101
Congestive heart failure	Elixhauser comorbidity software	Elixhauser comorbidity software
Dementia	29010, 29011, 29012, 29013, 29020, 29021, 2903, 29040, 29041, 29042, 29043, 2908, 2909, 2930, 2931, 2940, 2941, 29410, 29411, 29420, 29421	F0150, F0151, F0280, F0281, F0390, F0391, F04, F05, F070, F0781, F0789, F079, F09, F482, G300, G301, G308, G309, G3101, G3109, G311, G3183, R4181
Mechanical thrombectomy	ICD-9 procedural code 39.74	ICD-10 procedural codes 03CG3ZZ, 03CG3Z6, 03CG3Z7, 03CG4Z6, 03CG4ZZ, 03CH3ZZ, 03CH3Z7, 03CJ3ZZ, 03CJ3Z7, 03CK3ZZ, 03CK3Z7, 03CL3ZZ, 03CL3Z7, 03CP3ZZ, 03CP3Z7, 03CQ3ZZ and 03CQ3Z7
**Other CHA_2DS_2-VASc score component**		
Prior stroke	V1254	Z8673

Prior venous thromboembolism	V1251 and V1259	Z8671, Z86711 and Z86718
Prior myocardial infarction	412	I21, I22 and I252
Peripheral vascular disease	Elixhauser comorbidity software	Elixhauser comorbidity software

HCUP CCS stands for Healthcare Cost and Utilization Project Clinical Classification Software and can be accessed @ https://hcup-us.ahrq.gov/toolssoftware/ccs/AppendixASingleDX.txt

Elixhauser comorbidity components available at http://fmwww.bc.edu/repec/bocode/e/elixhauser.ado

Figure S1. Trends in the prevalence of atrial fibrillation in all admissions in the United States from 2010-2020 regardless of diagnoses

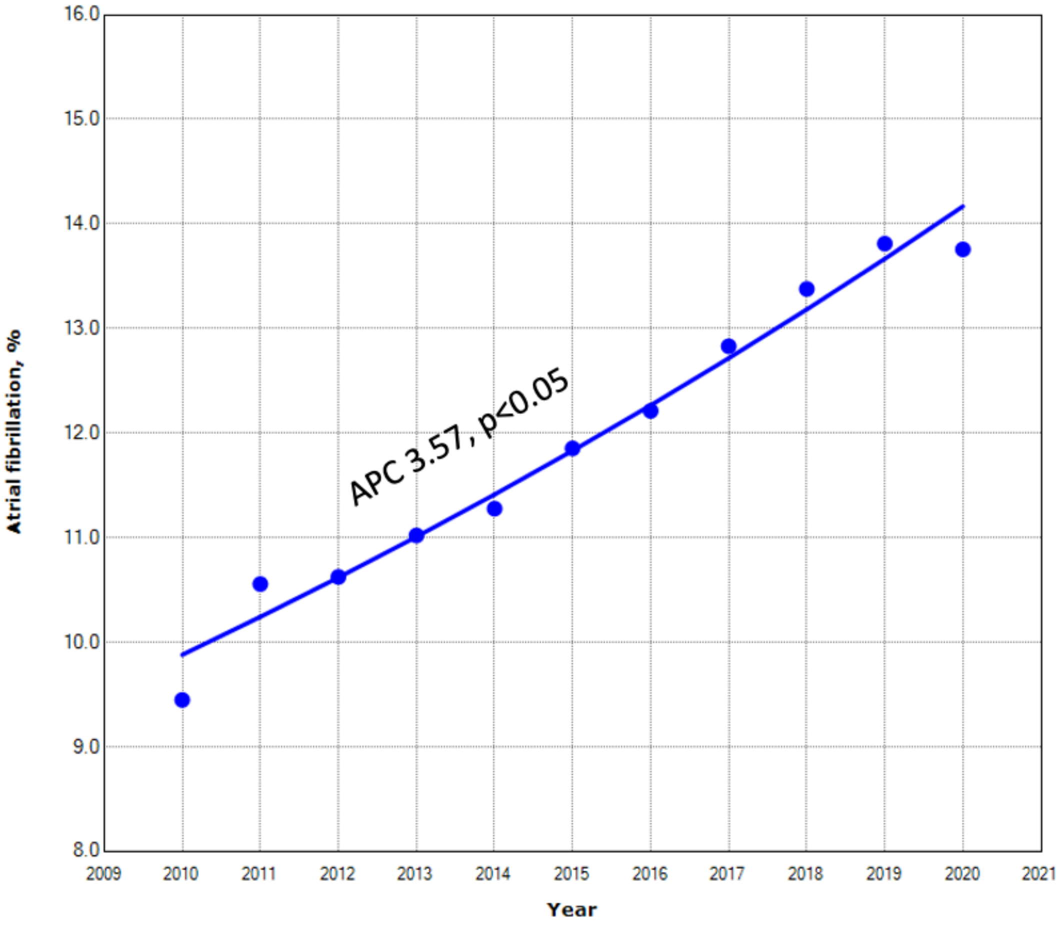

Figure S2. Trends in age- and sex-adjusted prevalence of atrial fibrillation in acute ischemic stroke admissions by race.

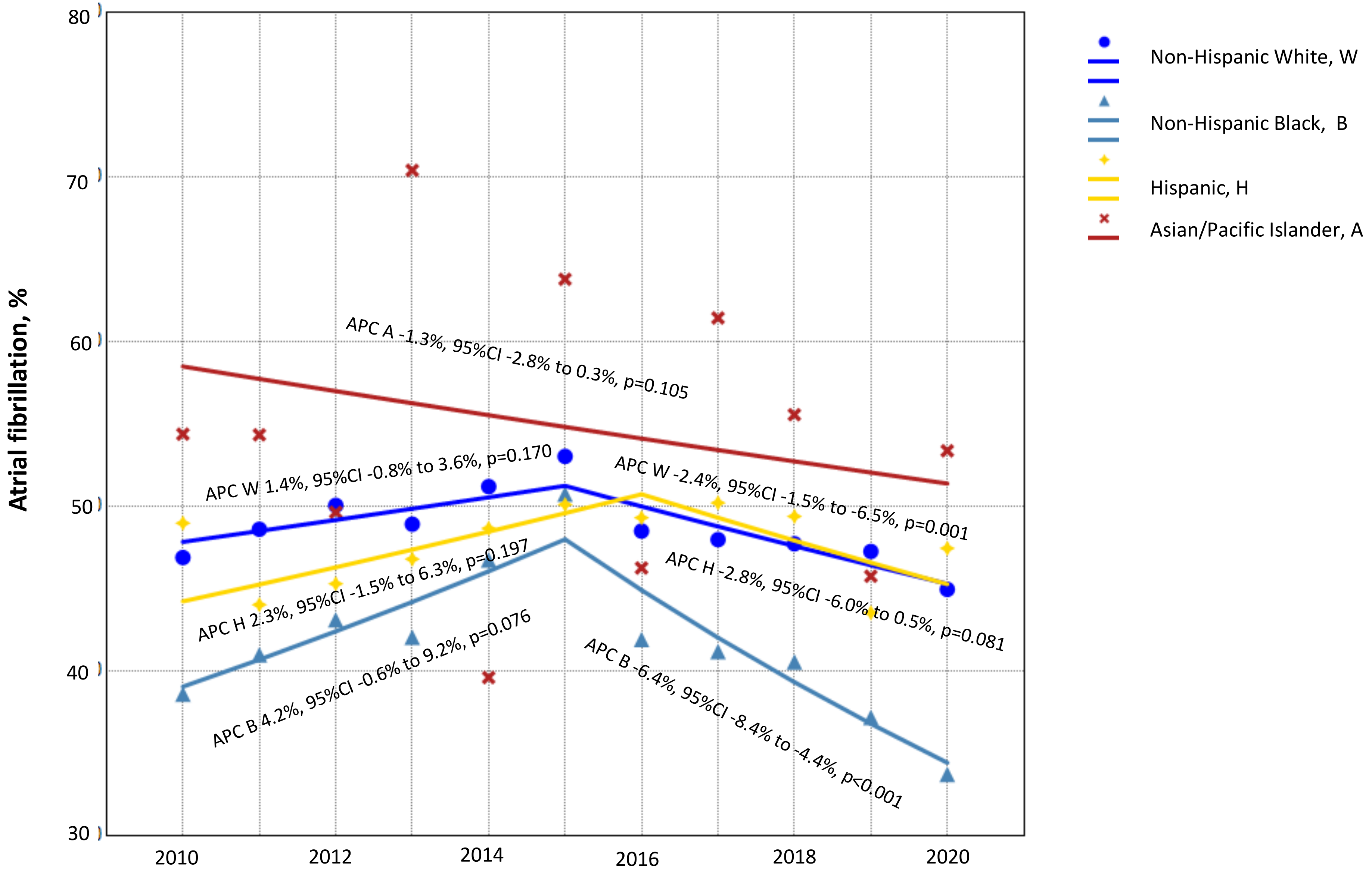

Supplement: Supplementary file 1 — Table S1. International Classification of Diseases Codes for identifying covariates. Figure S1. Trends in the prevalence of atrial fibrillation in all admissions in the United States from 2010‐2020 regardless of diagnoses Figure S2. Trends in age‐ and sex‐adjusted prevalence of atrial fibrillation in acute ischemic stroke admissions by race. [file SVI2-4-e001248-s001.pdf]
